# Supplementary material for: Magnetic order in 2D antiferromagnets revealed by spontaneous anisotropic magnetostriction
Source: Nat Commun. 2023 Dec 21;14:8503. doi: 10.1038/s41467-023-44180-4 (PMC10739885; doi:10.1038/s41467-023-44180-4)
Supplement: Supplementary file 1 — Supplementary Information [file 41467_2023_44180_MOESM1_ESM.pdf]

SUPPLEMENTARY INFORMATION: Magnetic order in 2D antiferromagnets revealed by spontaneous anisotropic magnetostriction

Maurits J. A. Houmes,[1,*] Gabriele Baglioni,[1,*] Makars Šiškins,[1,*] Martin Lee,[1] Dorye L. Esteras,[2] Alberto M. Ruiz,[2] Samuel Mañas-Valero,[1,2] Carla Boix-Constant,[2] Jose J. Baldoví,[2] Eugenio Coronado,[2] Yaroslav M. Blanter,[1] Peter G. Steeneken,[1,3] and Herre S. J. van der Zant[1]

[1]*Kavli Institute of Nanoscience, Delft University of Technology, Lorentzweg 1, 2628 CJ, Delft, The Netherlands*

[2]*Instituto de Ciencia Molecular (ICMol), Universitat de València, c/Catedrático José Beltrán 2, 46980 Paterna, Spain*

[3]*Department of Precision and Microsystems Engineering, Delft University of Technology, Mekelweg 2, 2628 CD, Delft, The Netherlands*

CONTENTS

Supplementary Note 1. Density Functional Theory calculations 2

Supplementary Note 2. Landau theory of second-order phase transitions and spontaneous magnetostriction 13
- Order parameter and critical exponent 13
- Magnetostrictive strain and resonance frequency 14

Supplementary Note 3. Derivation of anisotropic resonance frequency 15

Supplementary Note 4. Anisotropic resonance frequency of $FePS_3$ resonators 18

Supplementary Note 5. Order parameter related frequency difference $\tilde{f}_{\mathrm{b}}^2 - \tilde{f}_{\theta}^2$ 18

Supplementary Note 6. Critical curve fit 19

Supplementary References 21

* These authors contributed equally.

Supplementary Note 1. DENSITY FUNCTIONAL THEORY CALCULATIONS

This section summarizes the results of orbital-resolved magnetic exchange analyses based on maximally localized Wannier functions, for both the crystallographic and optimized structures. These results are included in Supplementary Tables 7 – 42. Supplementary Tables 43 – 48 display the hopping integrals with the S atoms (Supplementary Fig. 4) that mediate the super-exchange interactions between magnetic centers for each channel.

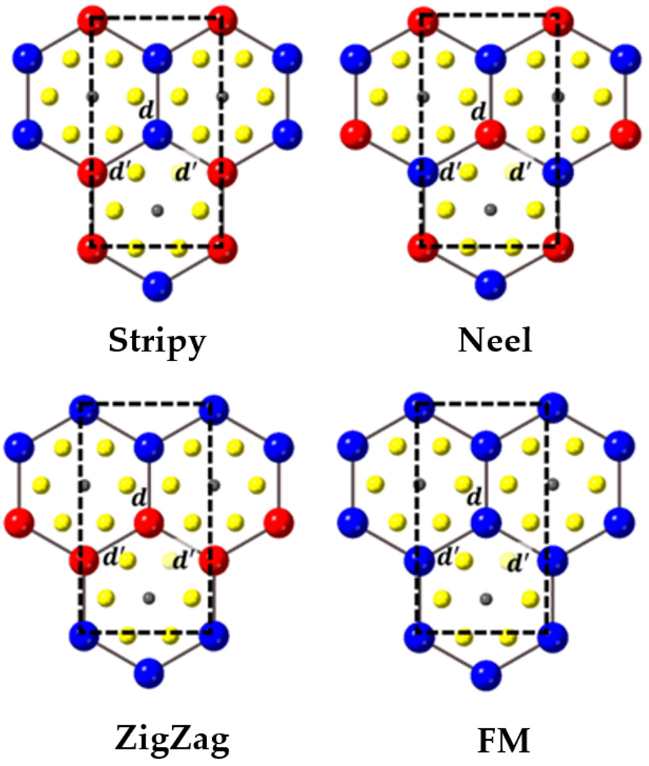

SUPPLEMENTARY FIG. 1. Top view of a single-layer of MPS_3 for different magnetic configurations, namely stripy, Néel, zigzag and ferromagnetic (FM). Blue and red balls represent transition metal atoms with spin up and down components, respectively.

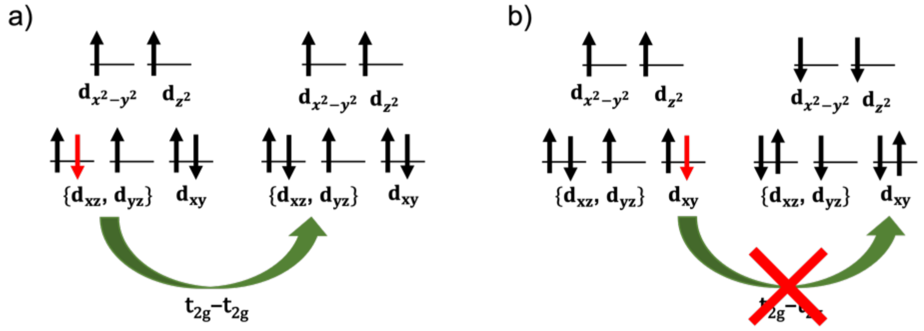

SUPPLEMENTARY FIG. 2. Electron configuration of the Co^{2+} magnetic ions connected by J_1 (a) and J_2 (b), showing parallel and antiparallel spin orientations, respectively. Green arrows illustrate the most relevant ferromagnetic superexchange channels, namely d_{yz}-d_{yz} and d_{xy}-d_{xy} (cancelled), for J_1 and J_2 respectively

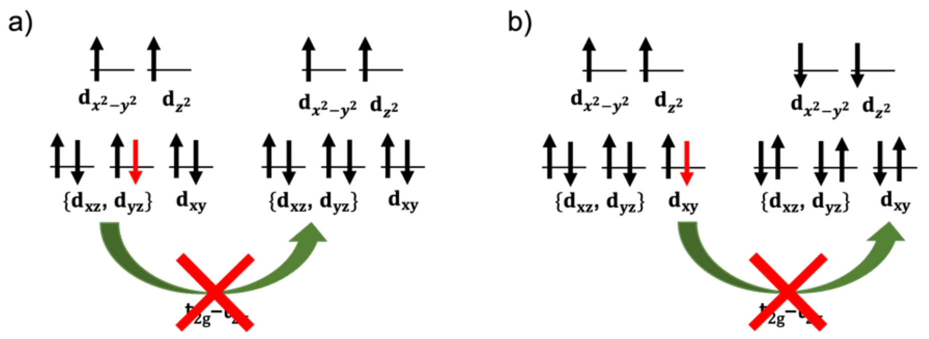

SUPPLEMENTARY FIG. 3. Electron configuration of the Ni^{2+} magnetic ions connected by J_1 (a) and J_2 (b), showing parallel and antiparallel spin orientations, respectively. Green arrows illustrate the most relevant ferromagnetic superexchange channels, namely d_{yz}-d_{yz} (cancelled) and d_{xy}-d_{xy} (cancelled), for J_1 and J_2 respectively.

SUPPLEMENTARY TABLE 1. $CoPS_3$, $FePS_3$ and $NiPS_3$ lattice parameters of the crystallographic non-magnetic (NM) and Stripy antiferromagnetic (AF) configurations

	**$CoPS_3$**		**$FePS_3$**		**$NiPS_3$**	
**Lattice parameter (Å)**	**a**	**b**	**a**	**b**	**a**	**b**
**Crystallographic NM**	5.895	10.190	5.947	10.301	5.812	10.070
**AF-stripy**	5.772	10.003	5.978	10.172	5.826	10.084
**Change (%)**	-2.086	-1.835	+0.521	-1.252	+0.241	+0.139

SUPPLEMENTARY TABLE 2. $CoPS_3$, $FePS_3$ and $NiPS_3$ lattice parameters of the crystallographic non-magnetic (NM) and Néel antiferromagnetic (AF) configurations.

	**$CoPS_3$**		**$FePS_3$**		**$NiPS_3$**	
**Lattice parameter (Å)**	**a**	**b**	**a**	**b**	**a**	**b**
**Crystallographic NM**	5.895	10.190	5.947	10.301	5.812	10.070
**AF-Néel**	5.885	10.187	5.947	10.303	5.834	10.106
**Change (%)**	-0.169	-0.029	+0.000	+0.019	+0.378	+0.357

SUPPLEMENTARY TABLE 3. $CoPS_3$, $FePS_3$ and $NiPS_3$ lattice parameters of the crystallographic non-magnetic (NM) and ferromagnetic (FM) configurations.

	**$CoPS_3$**		**$FePS_3$**		**$NiPS_3$**	
**Lattice parameter (Å)**	**a**	**b**	**a**	**b**	**a**	**b**
**Crystallographic NM**	5.895	10.190	5.947	10.301	5.812	10.070
**FM**	5.836	10.103	5.932	10.278	5.824	10.089
**Change (%)**	-1.001	-0.854	-0.252	-0.223	+0.207	+0.189

SUPPLEMENTARY TABLE 4. $CoPS_3$, $FePS_3$ and $NiPS_3$ distances (d and d') of the crystallographic non-magnetic (NM) and zigzag antiferromagnetic (AF) configurations.

	**$CoPS_3$**		**$FePS_3$**		**$NiPS_3$**	
**Distance (Å)**	**d**	**d'**	**d**	**d'**	**d**	**d'**
**Crystallographic NM**	3.397	3.402	3.433	3.433	3.357	3.356
**AF-zigzag**	3.723	3.192	3.626	3.316	3.353	3.359
**Change (%)**	+9.596	-6.172	+5.621	-3.408	-0.119	+0.089

SUPPLEMENTARY TABLE 5. $CoPS_3$, $FePS_3$ and $NiPS_3$ distances (d and d') of the crystallographic non-magnetic (NM) and Stripy antiferromagnetic (AF) configurations.

	**$CoPS_3$**		**$FePS_3$**		**$NiPS_3$**	
**Distance (Å)**	**d**	**d'**	**d**	**d'**	**d**	**d'**
**Crystallographic NM**	3.397	3.402	3.433	3.433	3.357	3.356
**AF-stripy**	3.337	3.332	2.670	3.843	3.373	3.357
**Change (%)**	-1.766	-2.058	-22.225	+11.943	+0.477	+0.029

SUPPLEMENTARY TABLE 6. $CoPS_3$, $FePS_3$ and $NiPS_3$ distances (d and d') of the crystallographic non-magnetic (NM) and Néel antiferromagnetic (AF) configurations.

	**$CoPS_3$**		**$FePS_3$**		**$NiPS_3$**	
**Distance (Å)**	**d**	**d'**	**d**	**d'**	**d**	**d'**
**Crystallographic NM**	3.397	3.402	3.433	3.433	3.357	3.356
**AF-Néel**	3.393	3.398	3.436	3.433	3.369	3.369
**Change (%)**	-0.118	-0.118	+0.087	+0.000	+0.357	+0.387

SUPPLEMENTARY TABLE 7. $CoPS_3$, $FePS_3$ and $NiPS_3$ distances (d and d') of the crystallographic non-magnetic (NM) and ferromagnetic (FM) configurations.

	**$CoPS_3$**		**$FePS_3$**		**$NiPS_3$**	
**Distance (Å)**	**d**	**d'**	**d**	**d'**	**d**	**d'**
**Crystallographic NM**	3.397	3.402	3.433	3.433	3.357	3.356
**FM**	3.368	3.368	3.429	3.424	3.363	3.362
**Change (%)**	-0.854	-0.999	+0.116	+0.262	+0.179	+0.179

SUPPLEMENTARY TABLE 8. Orbital resolved exchange parameters for the experimental structure of $FePS_3$.

**J_1 (meV)**	**d_{z2}**	**d_{xz}**	**d_{yz}**	**d_{x2y2}**	**d_{xy}**
**d_{z2}**	-0.467	-0.154	-0.157	-0.047	-0.027
**d_{xz}**	-0.154	7.091	0.063	-0.307	0.288
**d_{yz}**	-0.157	0.063	7.215	-0.103	0.109
**d_{x2y2}**	-0.047	-0.307	-0.103	-0.194	-0.017
**d_{xy}**	-0.027	0.288	0.109	-0.017	1.612

SUPPLEMENTARY TABLE 9. Orbital resolved exchange parameters for the experimental structure of $FePS_3$.

**J_2 (meV)**	**d_{z2}**	**d_{xz}**	**d_{yz}**	**d_{x2y2}**	**d_{xy}**
**d_{z2}**	-0.045	-0.013	-0.011	-0.008	-1.18
**d_{xz}**	-0.013	0.334	0.14	0.0	0.791
**d_{yz}**	-0.011	0.14	0.418	0.036	0.634
**d_{x2y2}**	-0.008	0.0	0.036	-0.783	0.086
**d_{xy}**	-1.18	0.791	0.634	0.086	11.016

SUPPLEMENTARY TABLE 10. Orbital resolved exchange parameters for the experimental structure of $FePS_3$.

**J_3 (meV)**	**d_{z2}**	**d_{xz}**	**d_{yz}**	**d_{x2y2}**	**d_{xy}**
**d_{z2}**	0.01	-0.058	-0.065	-0.028	-0.011
**d_{xz}**	-0.058	-0.02	-0.037	-0.101	-0.076
**d_{yz}**	-0.065	-0.037	-0.022	-0.122	0.089
**d_{x2y2}**	-0.028	-0.101	-0.122	-0.004	-0.041
**d_{xy}**	-0.011	-0.076	-0.089	-0.041	0.405

SUPPLEMENTARY TABLE 11. Orbital resolved exchange parameters for the experimental structure of $FePS_3$.

$\mathbf{J}_4$ (meV)	$\mathbf{d}_{z2}$	$\mathbf{d}_{xz}$	$\mathbf{d}_{yz}$	$\mathbf{d}_{x2y2}$	$\mathbf{d}_{xy}$
$\mathbf{d}_{z2}$	-0.121	0.002	0.003	0.013	-0.234
$\mathbf{d}_{xz}$	0.002	-0.093	-0.022	-0.005	-0.146
$\mathbf{d}_{yz}$	0.003	-0.022	-0.081	0.002	0.122
$\mathbf{d}_{x2y2}$	0.012	-0.005	0.002	-0.01	-0.18
$\mathbf{d}_{xy}$	-0.234	-0.146	-0.122	-0.18	-2.509

SUPPLEMENTARY TABLE 12. Orbital resolved exchange parameters for the experimental structure of $FePS_3$.

$\mathbf{J}_5$ (meV)	$\mathbf{d}_{z2}$	$\mathbf{d}_{xz}$	$\mathbf{d}_{yz}$	$\mathbf{d}_{x2y2}$	$\mathbf{d}_{xy}$
$\mathbf{d}_{z2}$	-2.323	0.019	0.015	-1.175	0.148
$\mathbf{d}_{xz}$	0.019	0.035	0.024	-0.01	-0.239
$\mathbf{d}_{yz}$	0.015	0.024	0.022	-0.032	0.154
$\mathbf{d}_{x2y2}$	-1.175	-0.01	-0.032	-0.154	-0.138
$\mathbf{d}_{xy}$	0.148	-0.239	-0.154	-0.138	-2.491

SUPPLEMENTARY TABLE 13. Orbital resolved exchange parameters for the experimental structure of $FePS_3$.

$\mathbf{J}_6$ (meV)	$\mathbf{d}_{z2}$	$\mathbf{d}_{xz}$	$\mathbf{d}_{yz}$	$\mathbf{d}_{x2y2}$	$\mathbf{d}_{xy}$
$\mathbf{d}_{z2}$	-0.156	0.03	0.025	-0.0	-0.05
$\mathbf{d}_{xz}$	0.03	-0.012	0.004	0.053	-0.073
$\mathbf{d}_{yz}$	0.025	0.004	-0.01	0.04	0.042
$\mathbf{d}_{x2y2}$	-0.0	0.053	0.04	-5.914	-0.044
$\mathbf{d}_{xy}$	-0.05	-0.073	-0.042	-0.044	0.325

SUPPLEMENTARY TABLE 14. Orbital resolved exchange parameters for the spin polarized optimized structure of $FePS_3$.

$\mathbf{J}_1$ (meV)	$\mathbf{d}_{z2}$	$\mathbf{d}_{xz}$	$\mathbf{d}_{yz}$	$\mathbf{d}_{x2y2}$	$\mathbf{d}_{xy}$
$\mathbf{d}_{z2}$	-0.899	-0.192	-0.188	-0.127	-0.017
$\mathbf{d}_{xz}$	-0.192	8.323	0.035	-0.29	0.306
$\mathbf{d}_{yz}$	-0.188	0.035	8.383	-0.088	0.12
$\mathbf{d}_{x2y2}$	-0.127	-0.29	-0.088	-0.28	-0.044
$\mathbf{d}_{xy}$	-0.017	0.306	0.12	-0.044	1.823

SUPPLEMENTARY TABLE 15. Orbital resolved exchange parameters for the spin polarized optimized structure of $FePS_3$.

$\mathbf{J}_2$ (meV)	$\mathbf{d}_{z2}$	$\mathbf{d}_{xz}$	$\mathbf{d}_{yz}$	$\mathbf{d}_{x2y2}$	$\mathbf{d}_{xy}$
$\mathbf{d}_{z2}$	-0.104	-0.021	-0.019	-0.009	-2.114
$\mathbf{d}_{xz}$	-0.021	0.161	0.019	-0.005	0.5
$\mathbf{d}_{yz}$	-0.019	0.019	0.182	0.002	0.432
$\mathbf{d}_{x2y2}$	-0.009	-0.005	0.002	0.128	0.015
$\mathbf{d}_{xy}$	-2.114	0.5	0.432	0.015	5.022

SUPPLEMENTARY TABLE 16. Orbital resolved exchange parameters for the spin polarized optimized structure of $FePS_3$.

$\mathbf{J}_3$ (meV)	$\mathbf{d}_{z2}$	$\mathbf{d}_{xz}$	$\mathbf{d}_{yz}$	$\mathbf{d}_{x2y2}$	$\mathbf{d}_{xy}$
$\mathbf{d}_{z2}$	0.012	-0.083	-0.093	-0.059	-0.013
$\mathbf{d}_{xz}$	-0.083	-0.025	-0.044	-0.088	-0.059
$\mathbf{d}_{yz}$	-0.093	-0.044	-0.027	-0.105	-0.064
$\mathbf{d}_{x2y2}$	-0.059	-0.088	-0.105	0.007	-0.038
$\mathbf{d}_{xy}$	-0.013	-0.059	-0.064	-0.038	0.278

SUPPLEMENTARY TABLE 17. Orbital resolved exchange parameters for the spin polarized optimized structure of $FePS_3$.

J_4 (meV)	d_{z2}	d_{xz}	d_{yz}	d_{x2y2}	d_{xy}
d_{z2}	-0.11	0.002	0.003	0.036	-0.212
d_{xz}	0.002	-0.112	-0.039	-0.014	-0.061
d_{yz}	0.003	-0.039	-0.105	0.0	-0.066
d_{x2y2}	0.036	-0.014	0.0	-0.004	-0.164
d_{xy}	-0.212	-0.061	-0.066	-0.164	-1.186

SUPPLEMENTARY TABLE 18. Orbital resolved exchange parameters for the spin polarized optimized structure of $FePS_3$.

J_5 (meV)	d_{z2}	d_{xz}	d_{yz}	d_{x2y2}	d_{xy}
d_{z2}	-2.613	-0.016	-0.014	-1.223	0.154
d_{xz}	-0.016	-0.008	0.032	-0.059	-0.105
d_{yz}	-0.014	0.032	-0.016	-0.075	-0.06
d_{x2y2}	-1.223	-0.059	-0.075	-0.181	-0.122
d_{xy}	0.154	-0.105	-0.06	-0.122	-1.114

SUPPLEMENTARY TABLE 19. Orbital resolved exchange parameters for the spin polarized optimized structure of $FePS_3$.

J_6 (meV)	d_{z2}	d_{xz}	d_{yz}	d_{x2y2}	d_{xy}
d_{z2}	-0.196	0.064	0.056	-0.001	-0.236
d_{xz}	0.064	-0.026	0.005	0.083	-0.092
d_{yz}	0.056	0.005	-0.022	0.083	-0.071
d_{x2y2}	-0.001	0.083	0.083	-5.386	-0.025
d_{xy}	-0.236	-0.092	-0.071	-0.025	1.036

SUPPLEMENTARY TABLE 20. Orbital resolved exchange parameters for the experimental structure of $CoPS_3$.

J_1 (meV)	d_{z2}	d_{xz}	d_{yz}	d_{x2y2}	d_{xy}
d_{z2}	0.168	-0.907	-0.922	0.108	-0.221
d_{xz}	-0.907	7.514	0.105	-0.977	-0.417
d_{yz}	-0.922	0.105	7.465	-1.266	-0.066
d_{x2y2}	0.108	-0.977	-1.266	-0.094	0.073
d_{xy}	-0.221	-0.417	-0.065	0.073	-0.36

SUPPLEMENTARY TABLE 21. Orbital resolved exchange parameters for the experimental structure of $CoPS_3$.

J_2 (meV)	d_{z2}	d_{xz}	d_{yz}	d_{x2y2}	d_{xy}
d_{z2}	-0.371	0.078	0.066	-0.002	-0.242
d_{xz}	0.078	0.524	0.022	0.115	-0.093
d_{yz}	0.066	0.022	0.54	-0.106	0.126
d_{x2y2}	-0.002	0.115	-0.106	-0.687	-0.034
d_{xy}	-0.242	-0.093	0.126	-0.034	-0.068

SUPPLEMENTARY TABLE 22. Orbital resolved exchange parameters for the experimental structure of $CoPS_3$.

J_3 (meV)	d_{z2}	d_{xz}	d_{yz}	d_{x2y2}	d_{xy}
d_{z2}	0.069	-0.117	-0.114	-0.047	-0.004
d_{xz}	-0.117	-0.06	-0.024	-0.326	-0.003
d_{yz}	-0.114	-0.024	-0.059	-0.367	0.01
d_{x2y2}	-0.047	-0.326	-0.367	-0.17	0.003
d_{xy}	-0.004	-0.003	0.01	0.004	-0.022

SUPPLEMENTARY TABLE 23. Orbital resolved exchange parameters for the experimental structure of $CoPS_3$.

$\mathbf{J_4}$ (meV)	$\mathbf{d}_{z2}$	$\mathbf{d}_{xz}$	$\mathbf{d}_{yz}$	$\mathbf{d}_{x2y2}$	$\mathbf{d}_{xy}$
$\mathbf{d}_{z2}$	-0.025	-0.011	-0.001	0.015	0.046
$\mathbf{d}_{xz}$	-0.011	-0.062	-0.004	0.002	0.047
$\mathbf{d}_{yz}$	-0.001	-0.004	-0.05	-0.006	0.046
$\mathbf{d}_{x2y2}$	0.015	0.002	-0.006	-0.082	-0.013
$\mathbf{d}_{xy}$	0.046	0.047	0.046	-0.013	0.055

SUPPLEMENTARY TABLE 24. Orbital resolved exchange parameters for the experimental structure of $CoPS_3$.

$\mathbf{J_5}$ (meV)	$\mathbf{d}_{z2}$	$\mathbf{d}_{xz}$	$\mathbf{d}_{yz}$	$\mathbf{d}_{x2y2}$	$\mathbf{d}_{xy}$
$\mathbf{d}_{z2}$	-3.482	-0.181	-0.163	-0.862	0.13
$\mathbf{d}_{xz}$	-0.181	-0.018	0.001	-0.263	0.034
$\mathbf{d}_{yz}$	-0.163	0.001	-0.044	-0.18	0.004
$\mathbf{d}_{x2y2}$	-0.862	-0.263	-0.18	-0.016	0.097
$\mathbf{d}_{xy}$	0.13	0.034	0.004	0.097	0.051

SUPPLEMENTARY TABLE 25. Orbital resolved exchange parameters for the experimental structure of $CoPS_3$.

$\mathbf{J_3}$ (meV)	$\mathbf{d}_{z2}$	$\mathbf{d}_{xz}$	$\mathbf{d}_{yz}$	$\mathbf{d}_{x2y2}$	$\mathbf{d}_{xy}$
$\mathbf{d}_{z2}$	-0.321	0.054	0.045	0.001	0.114
$\mathbf{d}_{xz}$	0.054	-0.018	-0.023	0.138	0.072
$\mathbf{d}_{yz}$	0.045	-0.023	-0.01	0.224	0.01
$\mathbf{d}_{x2y2}$	0.001	0.138	0.224	-3.745	-0.026
$\mathbf{d}_{xy}$	0.114	0.072	0.01	-0.026	0.029

SUPPLEMENTARY TABLE 26. Orbital resolved exchange parameters for the spin polarized optimized structure of $CoPS_3$.

$\mathbf{J_1}$ (meV)	$\mathbf{d}_{z2}$	$\mathbf{d}_{xz}$	$\mathbf{d}_{yz}$	$\mathbf{d}_{x2y2}$	$\mathbf{d}_{xy}$
$\mathbf{d}_{z2}$	-0.029	-0.889	-0.868	-0.449	-0.182
$\mathbf{d}_{xz}$	-0.889	10.043	0.01	-0.658	0.055
$\mathbf{d}_{yz}$	-0.868	0.01	10.011	-0.76	0.152
$\mathbf{d}_{x2y2}$	-0.449	-0.658	-0.76	0.024	-0.03
$\mathbf{d}_{xy}$	-0.182	0.055	0.152	-0.03	-0.173

SUPPLEMENTARY TABLE 27. Orbital resolved exchange parameters for the spin polarized optimized structure of $CoPS_3$.

$\mathbf{J_2}$ (meV)	$\mathbf{d}_{z2}$	$\mathbf{d}_{xz}$	$\mathbf{d}_{yz}$	$\mathbf{d}_{x2y2}$	$\mathbf{d}_{xy}$
$\mathbf{d}_{z2}$	-0.213	0.02	0.018	0.0	-0.113
$\mathbf{d}_{xz}$	0.02	0.106	-0.008	-0.014	0.122
$\mathbf{d}_{yz}$	0.018	-0.008	0.109	-0.038	0.128
$\mathbf{d}_{x2y2}$	0.0	-0.014	-0.038	-0.035	0.0
$\mathbf{d}_{xy}$	-0.113	0.122	0.128	0.0	-0.199

SUPPLEMENTARY TABLE 28. Orbital resolved exchange parameters for the spin polarized optimized structure of $CoPS_3$.

$\mathbf{J_3}$ (meV)	$\mathbf{d}_{z2}$	$\mathbf{d}_{xz}$	$\mathbf{d}_{yz}$	$\mathbf{d}_{x2y2}$	$\mathbf{d}_{xy}$
$\mathbf{d}_{z2}$	0.029	-0.135	-0.136	-0.162	-0.027
$\mathbf{d}_{xz}$	-0.135	-0.049	-0.018	-0.2	0.051
$\mathbf{d}_{yz}$	-0.136	-0.018	-0.049	-0.215	0.051
$\mathbf{d}_{x2y2}$	-0.162	-0.2	-0.215	-0.089	-0.007
$\mathbf{d}_{xy}$	-0.027	0.051	0.051	-0.007	0.011

SUPPLEMENTARY TABLE 29. Orbital resolved exchange parameters for the spin polarized optimized structure of $CoPS_3$.

$\mathbf{J}_4$ (meV)	$\mathbf{d}_{z2}$	$\mathbf{d}_{xz}$	$\mathbf{d}_{yz}$	$\mathbf{d}_{x2y2}$	$\mathbf{d}_{xy}$
$\mathbf{d}_{z2}$	-0.014	0.002	0.005	0.068	-0.048
$\mathbf{d}_{xz}$	0.002	-0.019	-0.027	-0.002	0.009
$\mathbf{d}_{yz}$	0.005	-0.027	-0.014	-0.013	0.016
$\mathbf{d}_{x2y2}$	0.068	-0.002	-0.013	-0.071	-0.021
$\mathbf{d}_{xy}$	-0.048	0.009	0.016	-0.021	0.002

SUPPLEMENTARY TABLE 30. Orbital resolved exchange parameters for the spin polarized optimized structure of $CoPS_3$.

$\mathbf{J}_5$ (meV)	$\mathbf{d}_{z2}$	$\mathbf{d}_{xz}$	$\mathbf{d}_{yz}$	$\mathbf{d}_{x2y2}$	$\mathbf{d}_{xy}$
$\mathbf{d}_{z2}$	-3.726	-0.232	-0.228	-1.283	0.014
$\mathbf{d}_{xz}$	-0.232	-0.055	0.0	-0.211	0.002
$\mathbf{d}_{yz}$	-0.229	0.0	-0.058	-0.183	-0.004
$\mathbf{d}_{x2y2}$	-1.283	-0.211	-0.183	-0.06	0.002
$\mathbf{d}_{xy}$	0.014	0.002	-0.004	0.002	0.002

SUPPLEMENTARY TABLE 31. Orbital resolved exchange parameters for the spin polarized optimized structure of $CoPS_3$.

$\mathbf{J}_6$ (meV)	$\mathbf{d}_{z2}$	$\mathbf{d}_{xz}$	$\mathbf{d}_{yz}$	$\mathbf{d}_{x2y2}$	$\mathbf{d}_{xy}$
$\mathbf{d}_{z2}$	-0.483	0.099	0.087	0.001	0.316
$\mathbf{d}_{xz}$	0.099	-0.068	0.0	0.08	0.064
$\mathbf{d}_{yz}$	0.087	0.0	-0.066	0.108	0.041
$\mathbf{d}_{x2y2}$	0.001	0.08	0.108	-3.258	-0.01
$\mathbf{d}_{xy}$	0.316	0.064	0.041	-0.01	0.196

SUPPLEMENTARY TABLE 32. Orbital resolved exchange parameters for the experimental structure of $NiPS_3$.

$\mathbf{J}_1$ (meV)	$\mathbf{d}_{z2}$	$\mathbf{d}_{xz}$	$\mathbf{d}_{yz}$	$\mathbf{d}_{x2y2}$	$\mathbf{d}_{xy}$
$\mathbf{d}_{z2}$	-0.055	-0.008	-0.012	-0.038	0.002
$\mathbf{d}_{xz}$	-0.008	0.014	-0.002	0.011	0.001
$\mathbf{d}_{yz}$	-0.012	-0.002	0.012	0.007	0.0
$\mathbf{d}_{x2y2}$	-0.038	0.011	0.007	-0.312	-0.004
$\mathbf{d}_{xy}$	0.003	0.001	0.0	-0.004	-0.002

SUPPLEMENTARY TABLE 33. Orbital resolved exchange parameters for the experimental structure of $NiPS_3$.

$\mathbf{J}_2$ (meV)	$\mathbf{d}_{z2}$	$\mathbf{d}_{xz}$	$\mathbf{d}_{yz}$	$\mathbf{d}_{x2y2}$	$\mathbf{d}_{xy}$
$\mathbf{d}_{z2}$	-0.161	-0.012	-0.01	0.0	-0.011
$\mathbf{d}_{xz}$	-0.012	-0.006	0.001	0.001	0.005
$\mathbf{d}_{yz}$	-0.01	0.001	-0.006	0.001	0.005
$\mathbf{d}_{x2y2}$	0.0	0.001	0.001	-0.1	-0.008
$\mathbf{d}_{xy}$	-0.011	0.005	0.005	-0.008	0.017

SUPPLEMENTARY TABLE 34. Orbital resolved exchange parameters for the experimental structure of $NiPS_3$.

$\mathbf{J}_3$ (meV)	$\mathbf{d}_{z2}$	$\mathbf{d}_{xz}$	$\mathbf{d}_{yz}$	$\mathbf{d}_{x2y2}$	$\mathbf{d}_{xy}$
$\mathbf{d}_{z2}$	0.015	-0.0	0.0	0.004	0.001
$\mathbf{d}_{xz}$	-0.0	-0.0	-0.001	0.011	-0.0
$\mathbf{d}_{yz}$	0.0	-0.001	-0.0	0.012	-0.0
$\mathbf{d}_{x2y2}$	0.004	0.011	0.012	0.053	0.007
$\mathbf{d}_{xy}$	0.001	-0.0	-0.0	0.007	-0.0

SUPPLEMENTARY TABLE 35. Orbital resolved exchange parameters for the experimental structure of $NiPS_3$.

$\mathbf{J}_4$ (meV)	$\mathbf{d}_{z2}$	$\mathbf{d}_{xz}$	$\mathbf{d}_{yz}$	$\mathbf{d}_{x2y2}$	$\mathbf{d}_{xy}$
$\mathbf{d}_{z2}$	0.204	-0.001	-0.0	0.024	0.003
$\mathbf{d}_{xz}$	-0.001	-0.001	0.0	-0.0	0.0
$\mathbf{d}_{yz}$	-0.0	0.0	-0.001	0.0	0.0
$\mathbf{d}_{x2y2}$	0.024	-0.0	0.0	-0.028	-0.009
$\mathbf{d}_{xy}$	0.003	0.0	0.0	-0.009	-0.001

SUPPLEMENTARY TABLE 36. Orbital resolved exchange parameters for the experimental structure of $NiPS_3$.

$\mathbf{J}_5$ (meV)	$\mathbf{d}_{z2}$	$\mathbf{d}_{xz}$	$\mathbf{d}_{yz}$	$\mathbf{d}_{x2y2}$	$\mathbf{d}_{xy}$
$\mathbf{d}_{z2}$	-5.191	-0.074	-0.061	-2.334	-0.042
$\mathbf{d}_{xz}$	-0.074	-0.006	-0.0	-0.031	0.0
$\mathbf{d}_{yz}$	-0.061	-0.0	-0.006	-0.024	-0.0
$\mathbf{d}_{x2y2}$	-2.334	-0.031	-0.024	-0.38	-0.003
$\mathbf{d}_{xy}$	-0.042	0.0	-0.0	-0.003	-0.001

SUPPLEMENTARY TABLE 37. Orbital resolved exchange parameters for the experimental structure of $NiPS_3$.

$\mathbf{J}_6$ (meV)	$\mathbf{d}_{z2}$	$\mathbf{d}_{xz}$	$\mathbf{d}_{yz}$	$\mathbf{d}_{x2y2}$	$\mathbf{d}_{xy}$
$\mathbf{d}_{z2}$	-0.058	-0.002	-0.002	0.0	0.028
$\mathbf{d}_{xz}$	-0.002	0.0	0.0	-0.003	0.0
$\mathbf{d}_{yz}$	-0.002	0.0	0.0	-0.004	0.0
$\mathbf{d}_{x2y2}$	0.0	-0.003	-0.004	-10.997	-0.092
$\mathbf{d}_{xy}$	0.028	0.0	0.0	-0.092	-0.008

SUPPLEMENTARY TABLE 38. Orbital resolved exchange parameters for the spin polarized optimized structure of $NiPS_3$.

$\mathbf{J}_1$ (meV)	$\mathbf{d}_{z2}$	$\mathbf{d}_{xz}$	$\mathbf{d}_{yz}$	$\mathbf{d}_{x2y2}$	$\mathbf{d}_{xy}$
$\mathbf{d}_{z2}$	0.099	0.009	0.007	-0.05	0.007
$\mathbf{d}_{xz}$	0.009	0.004	-0.002	0.044	-0.0
$\mathbf{d}_{yz}$	0.007	-0.002	0.002	0.038	-0.0
$\mathbf{d}_{x2y2}$	-0.05	0.044	0.038	-0.219	-0.003
$\mathbf{d}_{xy}$	0.007	-0.0	-0.0	-0.003	-0.004

SUPPLEMENTARY TABLE 39. Orbital resolved exchange parameters for the spin polarized optimized structure of $NiPS_3$.

$\mathbf{J}_2$ (meV)	$\mathbf{d}_{z2}$	$\mathbf{d}_{xz}$	$\mathbf{d}_{yz}$	$\mathbf{d}_{x2y2}$	$\mathbf{d}_{xy}$
$\mathbf{d}_{z2}$	-0.17	-0.009	-0.007	0.001	0.08
$\mathbf{d}_{xz}$	-0.009	-0.008	-0.002	0.007	0.005
$\mathbf{d}_{yz}$	-0.007	-0.002	-0.009	0.007	0.004
$\mathbf{d}_{x2y2}$	0.001	0.007	0.007	0.054	-0.004
$\mathbf{d}_{xy}$	0.08	0.005	0.004	-0.004	0.002

SUPPLEMENTARY TABLE 40. Orbital resolved exchange parameters for the spin polarized optimized structure of $NiPS_3$.

$\mathbf{J}_3$ (meV)	$\mathbf{d}_{z2}$	$\mathbf{d}_{xz}$	$\mathbf{d}_{yz}$	$\mathbf{d}_{x2y2}$	$\mathbf{d}_{xy}$
$\mathbf{d}_{z2}$	0.012	-0.002	-0.001	-0.013	0.001
$\mathbf{d}_{xz}$	-0.002	-0.001	-0.001	0.008	-0.0
$\mathbf{d}_{yz}$	-0.001	-0.001	-0.0	0.008	-0.001
$\mathbf{d}_{x2y2}$	-0.013	0.008	0.008	0.028	0.01
$\mathbf{d}_{xy}$	0.001	-0.0	-0.001	0.01	-0.001

SUPPLEMENTARY TABLE 41. Orbital resolved exchange parameters for the spin polarized optimized structure of $NiPS_3$.

$\mathbf{J}_4$ (meV)	$\mathbf{d}_{z2}$	$\mathbf{d}_{xz}$	$\mathbf{d}_{yz}$	$\mathbf{d}_{x2y2}$	$\mathbf{d}_{xy}$
$\mathbf{d}_{z2}$	0.116	0.002	0.002	0.028	-0.006
$\mathbf{d}_{xz}$	0.002	-0.002	0.0	0.0	0.0
$\mathbf{d}_{yz}$	0.002	0.0	-0.002	0.0	0.0
$\mathbf{d}_{x2y2}$	0.028	0.0	0.0	-0.028	-0.01
$\mathbf{d}_{xy}$	-0.006	0.0	0.0	-0.01	0.0

SUPPLEMENTARY TABLE 42. Orbital resolved exchange parameters for the spin polarized optimized structure of $NiPS_3$.

$\mathbf{J}_5$ (meV)	$\mathbf{d}_{z2}$	$\mathbf{d}_{xz}$	$\mathbf{d}_{yz}$	$\mathbf{d}_{x2y2}$	$\mathbf{d}_{xy}$
$\mathbf{d}_{z2}$	-4.91	-0.056	-0.046	-2.12	-0.032
$\mathbf{d}_{xz}$	-0.056	-0.009	0.0	-0.013	0.0
$\mathbf{d}_{yz}$	-0.046	0.0	-0.008	-0.007	-0.0
$\mathbf{d}_{x2y2}$	-2.12	-0.013	-0.007	-0.353	-0.003
$\mathbf{d}_{xy}$	-0.032	0.0	-0.0	-0.003	-0.001

SUPPLEMENTARY TABLE 43. Orbital resolved exchange parameters for the spin polarized optimized structure of $NiPS_3$.

$\mathbf{J}_6$ (meV)	$\mathbf{d}_{z2}$	$\mathbf{d}_{xz}$	$\mathbf{d}_{yz}$	$\mathbf{d}_{x2y2}$	$\mathbf{d}_{xy}$
$\mathbf{d}_{z2}$	-0.04	-0.002	-0.002	0.0	0.048
$\mathbf{d}_{xz}$	-0.002	0.0	0.0	-0.003	0.0
$\mathbf{d}_{yz}$	-0.002	0.0	0.0	-0.004	0.0
$\mathbf{d}_{x2y2}$	0.0	-0.003	-0.004	-10.334	-0.07
$\mathbf{d}_{xy}$	0.048	0.0	0.0	-0.07	-0.012

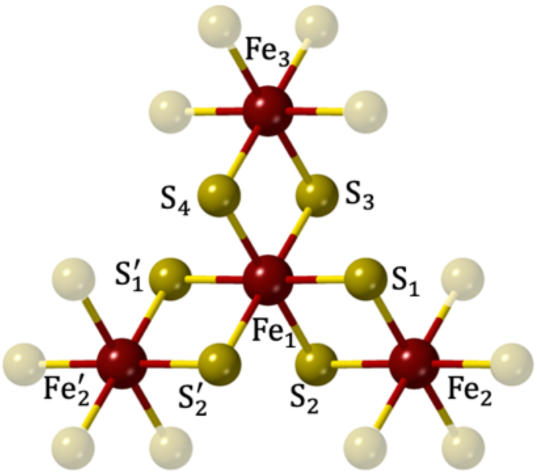

SUPPLEMENTARY FIG. 4. Top view of a cluster of single-layer $FePS_3$ centered in Fe_1 labelling the first Fe atoms neighbours and the S atoms that mediate super-exchange magnetic interactions.

SUPPLEMENTARY TABLE 44. Hopping parameters for the crystallographic structure of $FePS_3$.

		$S_1(p_z)$	$S_1(p_x)$	$S_1(p_y)$	$S_2(p_z)$	$S_2(p_x)$	$S_2(p_y)$
Fe_1	d_{z2}	1.089	-0.079	-0.243	0.054	-0.012	-0.608
	d_{xz}	-0.124	-0.331	-0.029	-0.041	-0.021	0.028
	d_{yz}	-0.254	-0.100	-0.357	-0.427	0.127	-0.002
	d_{x2y2}	0.002	-0.005	-0.037	0.127	-0.086	-0.987
	d_{xy}	0.003	0.021	0.038	0.037	-0.341	0.181
Fe_2	d_{z2}	-0.054	0.012	0.608	-1.089	0.079	0.243
	d_{xz}	0.041	0.021	-0.028	0.124	0.331	0.029
	d_{yz}	0.427	-0.127	0.002	0.254	0.100	0.357
	d_{x2y2}	-0.127	0.086	0.987	-0.002	0.005	0.037
	d_{xy}	-0.037	0.341	-0.181	-0.003	-0.021	-0.038

SUPPLEMENTARY TABLE 45. Hopping parameters for the crystallographic structure of $FePS_3$.

		$S_3(p_z)$	$S_3(p_x)$	$S_3(p_y)$	$S_4(p_z)$	$S_4(p_x)$	$S_4(p_y)$
Fe_1	d_{z2}	0.015	-0.525	-0.037	-0.015	0.040	0.525
	d_{xz}	-0.274	-0.023	-0.031	-0.020	-0.008	-0.029
	d_{yz}	0.037	0.030	0.010	0.276	0.030	0.026
	d_{x2y2}	-0.036	0.942	0.032	-0.028	0.078	0.939
	d_{xy}	-0.070	-0.043	-0.325	0.075	0.319	-0.078
Fe_3	d_{z2}	0.023	-0.056	-0.562	-0.022	0.562	0.053
	d_{xz}	0.028	0.018	0.062	0.334	-0.016	0.012
	d_{yz}	-0.336	-0.011	0.011	-0.049	-0.062	-0.018
	d_{x2y2}	0.041	-0.117	-1.048	0.052	-1.050	-0.058
	d_{xy}	-0.096	-0.414	0.080	0.090	0.056	0.423

SUPPLEMENTARY TABLE 46. Hopping parameters for the crystallographic structure of $CoPS_3$.

		$S_1(p_z)$	$S_1(p_x)$	$S_1(p_y)$	$S_2(p_z)$	$S_2(p_x)$	$S_2(p_y)$
Co_1	d_{z2}	1.019	-0.281	-0.178	0.032	-0.174	-0.581
	d_{xz}	-0.229	-0.339	0.098	-0.069	-0.032	0.039
	d_{yz}	-0.284	0.040	-0.341	-0.431	0.024	-0.056
	d_{x2y2}	-0.003	0.003	-0.043	0.059	-0.405	-0.902
	d_{xy}	-0.001	0.017	0.036	-0.089	-0.35	0.381
Co_2	d_{z2}	-0.032	0.174	0.581	-1.019	0.279	0.178
	d_{xz}	0.069	0.032	-0.039	0.229	0.339	-0.097
	d_{yz}	0.431	-0.025	0.056	0.284	-0.041	0.341
	d_{x2y2}	-0.059	0.406	0.901	0.003	-0.003	0.043
	d_{xy}	0.089	0.350	-0.382	0.001	-0.017	-0.036

SUPPLEMENTARY TABLE 47. Hopping parameters for the crystallographic structure of $CoPS_3$.

		$S_3(p_z)$	$S_3(p_x)$	$S_3(p_y)$	$S_4(p_z)$	$S_4(p_x)$	$S_4(p_y)$
Co_1	d_{z2}	0.017	-0.489	-0.033	-0.018	0.035	0.488
	d_{xz}	-0.241	-0.043	-0.014	-0.029	-0.013	-0.034
	d_{yz}	0.044	0.037	0.014	0.243	0.014	0.046
	d_{x2y2}	-0.042	0.904	0.011	-0.034	0.051	0.908
	d_{xy}	-0.072	-0.094	-0.299	0.077	0.296	-0.019
Co_3	d_{z2}	0.015	-0.031	-0.534	-0.015	0.534	0.029
	d_{xz}	0.037	0.020	0.056	0.282	0.011	0.015
	d_{yz}	-0.285	-0.015	-0.015	-0.054	-0.057	-0.020
	d_{x2y2}	0.037	-0.036	-1.011	0.046	-1.005	0.009
	d_{xy}	-0.089	-0.341	0.012	0.083	0.113	0.341

SUPPLEMENTARY TABLE 48. Hopping parameters for the crystallographic structure of $NiPS_3$.

		$\mathbf{S_1(p_z)}$	$\mathbf{S_1(p_x)}$	$\mathbf{S_1(p_y)}$	$\mathbf{S_2(p_z)}$	$\mathbf{S_2(p_x)}$	$\mathbf{S_2(p_y)}$
$\mathbf{Ni_1}$	$\mathbf{d}_{z2}$	1.023	-0.279	-0.112	0.009	-0.116	-0.553
	$\mathbf{d}_{xz}$	-0.288	-0.316	0.066	-0.055	-0.038	0.047
	$\mathbf{d}_{yz}$	-0.166	0.053	-0.352	-0.367	-0.011	0.015
	$\mathbf{d}_{x2y2}$	0.032	0.025	-0.022	0.044	-0.237	-0.890
	$\mathbf{d}_{xy}$	-0.017	-0.008	0.053	-0.057	-0.320	0.262
$\mathbf{Ni_2}$	$\mathbf{d}_{z2}$	-0.008	0.118	0.553	-1.026	0.275	0.113
	$\mathbf{d}_{xz}$	0.055	0.038	-0.047	0.288	0.316	-0.065
	$\mathbf{d}_{yz}$	0.366	0.009	-0.015	0.166	-0.055	0.353
	$\mathbf{d}_{x2y2}$	-0.043	0.241	0.889	-0.032	-0.025	0.022
	$\mathbf{d}_{xy}$	0.058	0.320	-0.263	0.017	0.008	-0.053

SUPPLEMENTARY TABLE 49. Hopping parameters for the crystallographic structure of $NiPS_3$.

		$\mathbf{S_3(p_z)}$	$\mathbf{S_3(p_x)}$	$\mathbf{S_3(p_y)}$	$\mathbf{S_4(p_z)}$	$\mathbf{S_4(p_x)}$	$\mathbf{S_4(p_y)}$
$\mathbf{Ni_1}$	$\mathbf{d}_{z2}$	0.034	-0.526	-0.061	-0.035	0.064	0.525
	$\mathbf{d}_{xz}$	-0.331	-0.053	-0.030	-0.011	-0.016	-0.031
	$\mathbf{d}_{yz}$	0.039	0.036	0.019	0.333	0.030	0.057
	$\mathbf{d}_{x2y2}$	-0.069	0.971	0.090	-0.057	0.160	0.955
	$\mathbf{d}_{xy}$	-0.068	0.003	-0.389	0.080	0.369	-0.170
$\mathbf{Ni_3}$	$\mathbf{d}_{z2}$	0.032	-0.05	-0.556	-0.031	0.557	0.047
	$\mathbf{d}_{xz}$	0.015	0.017	0.042	0.352	0.032	0.043
	$\mathbf{d}_{yz}$	-0.355	-0.042	-0.036	-0.045	-0.045	-0.020
	$\mathbf{d}_{x2y2}$	0.051	-0.140	-1.025	0.062	-1.036	-0.067
	$\mathbf{d}_{xy}$	-0.082	-0.393	0.159	0.071	0.019	0.408

Supplementary Note 2. LANDAU THEORY OF SECOND-ORDER PHASE TRANSITIONS AND SPONTANEOUS MAGNETOSTRICTION

Magnetostriction is a coupling between magnetic and mechanical parts of our system. This coupling can be described by an energy term in the total free energy of our system [1]. We can then write the total free energy as

$$F - F_0 = U_{\mathrm{el}}(z) + a(T - T_{\mathrm{N}})^{2\beta} L_i L_i + B L_i L_i L_i L_i - \sigma_{ij}(z)\lambda_{ijkl} L_k L_l. \tag{1}$$

Here F is the total free energy of in the AF phase at zero magnetic field, F_0 is the free energy of paramagnetic phase, $U_{\mathrm{el}}(z)$ is the elastic energy of a membrane with deflection z at its centre, T is the temperature of our system and T_{N} is the Néel temperature, L_i are the components of the Néel vector, β is a critical exponent, a and B are positive constants, $\sigma_{ij}(z)$ is the stress tensor and λ_{ijkl} is the magnetostriction tensor. The last term couples the stress to the Néel vector thereby describing the magnetostriction. If we assume the Néel vector to be fully aligned with the easy axis, Eq. (1) simplifies to:

$$F - F_0 = U_{\mathrm{el}}(z) + a(T - T_{\mathrm{N}})^{2\beta} L^2 + B L^4 - \sigma_{ij}(z)\lambda_{ij} L^2, \tag{2}$$

where L is the magnetic order parameter (i.e. the magnitude of the Néel vector). For notational convenience we write λ_{ij} in dropping the third and fourth index of λ_{ijkl} as only the component where kl corresponds to the easy axis contributes. The elastic energy in a homogeneous membrane is given by [2]

$$U_{\mathrm{el}} = \int\int \frac{S_{ijkl}}{2} \sigma_{ij}(x,y,z)\sigma_{kl}(x,y,z) dxdy, \tag{3}$$

where the integration runs over the in plane dimensions of the membrane, z is the membrane deflection at its centre and should not be confused with the out of plane coordinate. For ease of notation we will not explicitly write the integration and coordinate dependence from here on. Assuming the membrane thickness does not vary significantly we can take the out of plane component stress component to vanish, $\sigma_{zx} = \sigma_{zy} = \sigma_{zz} = 0$. Eq. (3) then simplifies to

$$U_{\mathrm{el}} = \frac{S_{xxxx}}{2}\sigma_{xx}\sigma_{xx} + \frac{S_{yyyy}}{2}\sigma_{yy}\sigma_{yy} + S_{xxyy}\sigma_{xx}\sigma_{yy} + S_{xxxy}\sigma_{xx}\sigma_{xy} + S_{yyxy}\sigma_{yy}\sigma_{xy} + \frac{S_{xyxy}}{2}\sigma_{xy}\sigma_{xy}. \tag{4}$$

Taking our coordinates such that x, y correspond with the principle stress directions all terms containing σ_{xy} vanish. This simplifies the elastic energy further to

$$U_{\mathrm{el}} = \frac{S_{xxxx}}{2}\sigma_{xx}\sigma_{xx} + \frac{S_{yyyy}}{2}\sigma_{yy}\sigma_{yy} + S_{xxyy}\sigma_{xx}\sigma_{yy}. \tag{5}$$

Assuming the material has isotropic elastic properties the relevant compliance tensor components are

$$S_{xxxx} = S_{yyyy} = \frac{1}{E} \text{ and } S_{xxyy} = \frac{-\nu}{E}. \tag{6}$$

Substituting this in to Eq. (5) we find

$$U_{\mathrm{el}} = \frac{1}{2E}\sigma_{xx}\sigma_{xx} + \frac{1}{2E}\sigma_{yy}\sigma_{yy} - \frac{\nu}{E}\sigma_{xx}\sigma_{yy}. \tag{7}$$

By taking the derivative of the free energy with respect to either z or L we find the forces acting on these degrees of freedom, ϕ_L and ϕ_z respectively to be given by

$$-\phi_L = \frac{d(F - F_0)}{dL} = 2a(T - T_{\mathrm{N}})^{2\beta} L + 4BL^3 - 2\sigma_{ij}(z)\lambda_{ij} L, \tag{8}$$

$$-\phi_z = \frac{d(F - F_0)}{dz} = \left(S_{ijkl}\sigma_{ij}(z) + \lambda_{kl} L^2\right)\frac{d\sigma_{kl}(z)}{dz}. \tag{9}$$

Order parameter and critical exponent

In order to find an equation that describes the order parameter as a function of temperature, we find a solution for equation (8) for the case where $\phi_L = 0$. Aside from the trivial solution $L = 0$, we find for below the transition the additional solution:

$$L^2 = -\frac{a}{2B}(T - T_{\mathrm{N}})^{2\beta} + \frac{\sigma_{ij}\lambda_{ij}}{2B}, \tag{10}$$

which could be rewritten for $T_{\rm N}^{*} = T_{\rm N} - (\frac{\sigma_{ij}\lambda_{ij}}{a})^{\frac{1}{2\beta}}$ [3] as:

$$L^2 = \frac{a}{2B}(T_{\rm N}^{*} - T)^{2\beta}. \tag{11}$$

This equation now describes the temperature dependence of the order parameter in a critical region near $T_{\rm N}$ with a corresponding critical exponent β.

Magnetostrictive strain and resonance frequency

To assess the magnetostriction contribution to strain and thus the frequency of a rectangular membrane resonator, we need to find stiffness of the membrane from its force-deflection equation. In doing that we analyse equation (9). First, we describe strain equation for the rectangular membrane at its centre as:

$$\epsilon_{xx}(z) = \epsilon_{0,x} + \frac{c_1}{2}\frac{z^2}{l^2} \tag{12a}$$

$$\epsilon_{yy}(z) = \epsilon_{0,y} + \frac{c_1}{2}\frac{z^2}{w^2} \tag{12b}$$

where c_1 is a geometrical pre-factor that describes the deflection shape of the fundamental mode of vibration [4, 5]. For $w \ll l$ we can neglect the z dependence of $\epsilon_{xx}(z)$. Now, we substitute (12) to (9), using the relation $\sigma_{ij} = C_{ijkl}\epsilon_{kl}$, we find

$$-\phi_z = \left(\frac{E}{1-\nu^2}\epsilon_{0,y} + \frac{\nu E}{1-\nu^2}\epsilon_{0,x} - \lambda_{yy}L^2\right)\frac{c_1}{w^2}z + \frac{E}{1-\nu^2}\frac{c_1^2}{2}\frac{z^3}{w^4}, \tag{13}$$

where we used that $C_{xxxx} = C_{yyyy} = \frac{E}{1-\nu^2}$ and $C_{yyxx} = C_{xxyy} = \frac{\nu E}{1-\nu^2}$. Eq. (13) becomes

$$-\phi_z = k_1 z - \frac{\lambda_{ij}c_1}{w^2}L^2 z + k_3 z^3 \tag{14}$$

where k_1 is the elastic linear stiffness and k_3 is the cubic elastic stiffness, given by

$$k_1 = \frac{E}{1-\nu^2}(\epsilon_{0,y} + \nu\epsilon_{0,x})\frac{c_1}{w^2} \tag{15}$$

$$k_3 = \frac{E}{1-\nu^2}\frac{c_1^2}{2w^4}. \tag{16}$$

Assuming small deflections we can neglect the z^3 contribution in eq. (13) and find that the linear stiffness is changed with respect to the purely elastic case. If we consider a rectangular cavity with it's long axis is parallel to the crystalline axis b or a respectively we find:

$$-\phi_{z\ \rm b,a} = \left(k_1 - \frac{c_1}{w^2}\lambda_{\rm a,b}L^2\right)z\,, \tag{17}$$

where $\lambda_{\rm a,b}$ are the phenomenological magnetostriction coefficients, chosen such that to couple a and b crystalline directions and L. That leads to a change in the effective linear stiffness $k_{b,a}$:

$$k_{\rm b,a} = k_1 - \frac{c_1}{w^2}\lambda_{\rm a,b}L^2, \tag{18}$$

which can be used to write down the frequency equations using $f_{\rm a,b} = \frac{1}{2\pi}\sqrt{\frac{k_{\rm a,b}}{m}}$,

$$f_{\rm b} \approx \frac{1}{2\pi}\sqrt{\frac{1}{m}\frac{c_1}{w^2}\left[\frac{E}{1-\nu^2}(\epsilon_{0,\rm a} + \nu\epsilon_{0,\rm b}) - \lambda_{\rm a}L^2\right]}, \tag{19a}$$

$$f_{\rm a} \approx \frac{1}{2\pi}\sqrt{\frac{1}{m}\frac{c_1}{w^2}\left[\frac{E}{1-\nu^2}(\epsilon_{0,\rm b} + \nu\epsilon_{0,\rm a}) - \lambda_{\rm b}L^2\right]}, \tag{19b}$$

where m is the mass of the membrane. And define magnetostrictive strain by:

$$\epsilon_{\mathrm{ms,a}} = \frac{c_1}{mw^2}\lambda_{\mathrm{a}}L^2, \tag{20}$$

$$\epsilon_{\mathrm{ms,b}} = \frac{c_1}{mw^2}\lambda_{\mathrm{b}}L^2. \tag{21}$$

Taking a difference of the squares of equations (19) and assuming $\epsilon_{0,\mathrm{a}} = \epsilon_{0,\mathrm{b}}$, we arrive at the final equation:

$$f_{\mathrm{b}}^2 - f_{\mathrm{a}}^2 = -\frac{1}{4\pi^2}\frac{c_1}{mw^2}\left[\lambda_{\mathrm{b}} - \lambda_{\mathrm{a}}\right]L^2, \tag{22}$$

which relates antiferromagnetic order parameter L and measured resonance frequencies of orthogonal resonators aligned to crystalline axes $f_{\mathrm{a,b}}$ in ordered phase. Finally, one can show that by plugging equation (11) to (22):

$$f_{\mathrm{b}}^2 - f_{\mathrm{a}}^2 \propto (T_{\mathrm{N}}^* - T)^{2\beta}, \tag{23}$$

which could be used to fit experimental data to extract critical exponent β near the phase transition temperature T_{N}^*.

Supplementary Note 3. DERIVATION OF ANISOTROPIC RESONANCE FREQUENCY

Here, we derive the general equation of the resonance frequency of a rectangular cavity oriented at an angle θ with respect to the crystalline axes, as schematically shown in Fig. 5. The global coordinate system is defined by the crystallographic axes a and b, along which the material deforms resulting in stresses σ_{aa} and σ_{bb}. The longest side of the cavity, with length l, can be oriented at an arbitrary angle θ with respect to b.

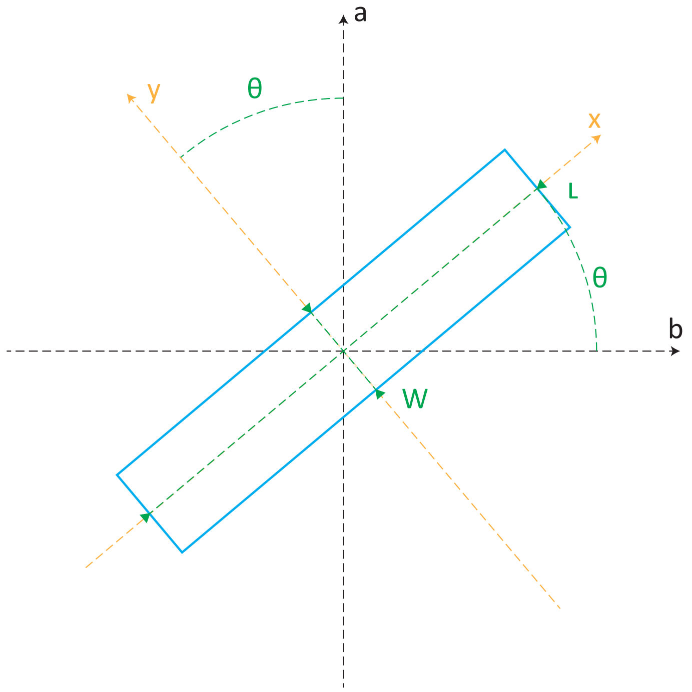

SUPPLEMENTARY FIG. 5. **Schematic illustration of rectangular membrane** Rectangular membrane of width w and length l oriented with its long side at an angle θ with respect to the crystalline direction b. The x-y direction refer to the main directions of the rectangular membrane.

Let us first consider a cavity oriented parallel to a crystallographic axis. Since the membranes are very thin, we can assume that the stress in the direction perpendicular to the plane is zero, $\sigma_{cc} = 0$, the membrane's stress tensor can be expressed as

$$\boldsymbol{\sigma} = \begin{pmatrix} \sigma_{aa} & \sigma_{ab} \\ \sigma_{ba} & \sigma_{bb} \end{pmatrix}_{ab}, \tag{24}$$

where the subscript $()_{ab}$ indicates we expressed the stress tensor in the basis of the crystallographic coordinate system. If we assume that there are no shear forces acting on the crystal lattice, $\sigma_{ab} = \sigma_{ba} = 0$, there will be no shear on cavities oriented along the main crystallographic axes. Now, if we consider a rectangular cavity rotated by θ with respect to the crystallographic axes, we can define a rotated xy-coordinate system oriented along the main axis of the rectangle. To express $\boldsymbol{\sigma}$ in this coordinate system we use the tensor transformation rule, $\sigma'_{ij} = q_{ki}q_{lj}\sigma_{kl}$ where q_{ij} are components of the rotation tensor transforming the ab-coordinate system, $\boldsymbol{e}$, into the xy-coordinate system, $\boldsymbol{e'}$ as $\boldsymbol{e}'_i = q_{ij}\boldsymbol{e}_j$. We then get:

$$\boldsymbol{\sigma} = \begin{pmatrix} \sigma_{xx} & \sigma_{xy} \\ \sigma_{yx} & \sigma_{yy} \end{pmatrix}_{xy} = \begin{pmatrix} \cos^2(\theta)\sigma_{aa} + \sin^2(\theta)\sigma_{bb} & -\cos(\theta)\sin(\theta)\sigma_{aa} + \sin(\theta)\cos(\theta)\sigma_{bb} \\ -\sin(\theta)\cos(\theta)\sigma_{aa} + \sin(\theta)\cos(\theta)\sigma_{bb} & \sin^2(\theta)\sigma_{aa} + \cos^2(\theta)\sigma_{bb} \end{pmatrix}_{xy}, \tag{25}$$

Then, the fundamental resonance frequency of a rectangular membrane oriented at an angle θ with respect to the crystallographic axis can be expressed as

$$f_\theta \approx \frac{1}{2}\sqrt{\frac{1}{\rho}\left(\frac{\sigma_{xx}}{l^2} + \frac{\sigma_{yy}}{w^2}\right)}. \tag{26}$$

In the case of high-aspect ratio membranes ($w \ll l$), Eq. 26 can be approximated to

$$f_\theta \approx \frac{1}{2}\sqrt{\frac{1}{\rho}\frac{\sigma_{yy}}{w^2}} = \frac{1}{2}\sqrt{\frac{1}{\rho w^2}\left(\sin^2(\theta)\sigma_{aa} + \cos^2(\theta)\sigma_{bb}\right)}, \tag{27}$$

which is Eq. 3 of the main text.

Now, let us consider the constitutive equations of the material:

$$c_1 = E(\epsilon_{\mathrm{fab},aa} - \epsilon_{\mathrm{th},aa} - \epsilon_{\mathrm{ms},aa}) = E\left(\epsilon_{\mathrm{fab},aa} - \int_{T_0}^{T_1} \alpha_{\mathrm{a}}(T)dT - \lambda_{\mathrm{a}}L^2(T_1)\right) = \sigma_{aa}(T_1) - \nu\sigma_{bb}(T_1) \tag{28}$$

$$c_2 = E(\epsilon_{\mathrm{fab},bb} - \epsilon_{\mathrm{th},bb} - \epsilon_{\mathrm{ms},bb}) = E\left(\epsilon_{\mathrm{fab},bb} - \int_{T_0}^{T_1} \alpha_{\mathrm{b}}(T)dT - \lambda_{\mathrm{b}}L^2(T_1)\right) = \sigma_{bb}(T_1) - \nu\sigma_{aa}(T_1), \tag{29}$$

where ϵ_{fab} is residual fabrication strain at $T = T_0$, ϵ_{th} and ϵ_{ms} are respectively the thermal expansion and magnetostriction contributions to strain, α is the thermal expansion coefficient, λ the magnetostriction coefficient and E is the Young's modulus, which is assumed to be isotropic. We can thus write

$$\sigma_{aa} = c_1 + \nu\sigma_{bb} \tag{30}$$

$$\sigma_{bb} = c_2 + \nu\sigma_{aa}, \tag{31}$$

which can be combined in the following expressions for σ_{aa} and σ_{bb}:

$$\sigma_{aa} = \frac{c_1 + \nu c_2}{1 - \nu^2} \tag{32}$$

$$\sigma_{bb} = \frac{c_2 + \nu c_1}{1 - \nu^2}. \tag{33}$$

We can now rewrite Eq. 27 in terms of the different contributions to strain, i.e. residual strain from fabrication (ϵ_{fab}), thermal expansion ($\propto \alpha$) and magnetostriction ($\propto \lambda$):

$$f_\theta(T) = \frac{1}{2}\sqrt{\frac{E}{\rho w^2(1-\nu^2)}\left[\sin^2\theta(c_1 + \nu c_2) + \cos^2\theta(c_2 + \nu c_1)\right]} \tag{34}$$

$$= \frac{1}{2}\sqrt{\frac{E}{\rho w^2(1-\nu^2)}[(\sin^2\theta + \nu\cos^2\theta)(\epsilon_{\mathrm{fab},aa} - \epsilon_{\mathrm{th},aa} - \epsilon_{\mathrm{ms},aa}) + (\cos^2\theta + \nu\sin^2\theta)(\epsilon_{\mathrm{fab},bb} - \epsilon_{\mathrm{th},bb} - \epsilon_{\mathrm{ms},bb})]}. \tag{35}$$

Which is consistent with Eq. (19). We can eliminate the pretension ϵ_{fab} terms by considering $\tilde{f}_\theta^{\,2}(T) = f_\theta^2(T) - f_\theta^2(T_0)$. In the following, we assume that the only anisotropic temperature-dependent contribution to the total strain comes

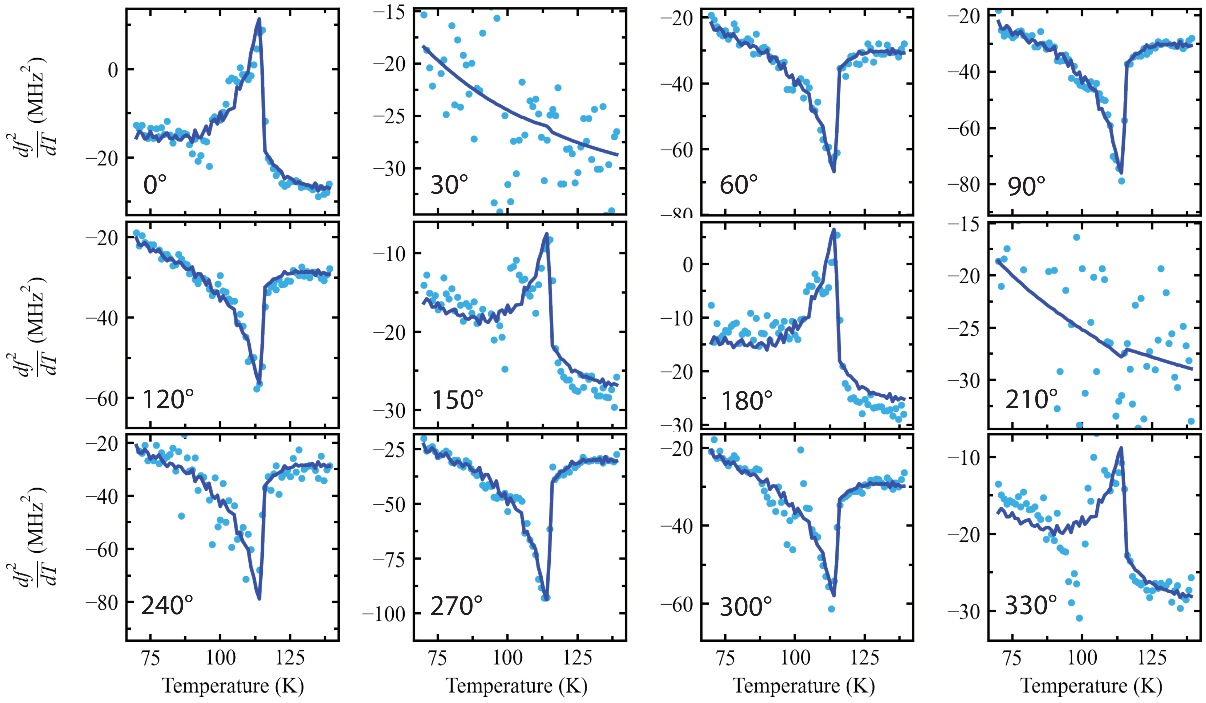

SUPPLEMENTARY FIG. 6. **Angle-resolved** $\frac{df^2}{dT}$**:** Plot of measured (light blue dots) $\frac{df^2}{dT}$ and fit to $b_1 C_{\mathrm{Debye}} + b_2 \frac{dL^2}{dT}$ (blue full line) for all angles of a star-shaped array of $CoPS_3$.

from magnetostriction, thus we take $\epsilon_{\mathrm{th},aa} = \epsilon_{\mathrm{th},bb} = \epsilon_{\mathrm{th}}$. By definition of θ we have $b \to \theta = 0°$ and $a \to \theta = 90°$. From Eq. (35), we then find that $\tilde{f}_{\mathrm{a}}^2 - \tilde{f}_{\mathrm{b}}^2$ becomes

$$\tilde{f}_{\mathrm{a}}^2 - \tilde{f}_{\mathrm{b}}^2 = \frac{E}{4\rho w^2(1+\nu)}\big(- \epsilon_{\mathrm{ms},aa} + \epsilon_{\mathrm{ms},bb}\big) \tag{36}$$

$$= -\frac{E}{4\rho w^2(1+\nu)}(\lambda_{\mathrm{a}} - \lambda_{\mathrm{b}})L^2 \tag{37}$$

from which we can directly extract the order parameter. The thermal expansion contribution to strain ϵ_α is proportional to the integral over the temperature of the thermal expansion coefficient α, which is proportional to the Debye specific heat, C_{Debye}, via the Grünesen parameter. Thus, the derivative with respect to temperature of f_θ^2

$$\frac{df_\theta^2}{dT} = \frac{E}{4\rho w^2(1-\nu^2)}\left[(\sin^2\theta + \nu\cos^2\theta)\left(-\alpha - \lambda_{\mathrm{a}}\frac{dL^2}{dT}\right) + (\cos^2\theta + \nu\sin^2\theta)\left(-\alpha - \lambda_{\mathrm{b}}\frac{dL^2}{dT}\right)\right] \tag{38}$$

$$= \frac{-E}{4\rho w^2(1-\nu^2)}\left[\alpha(1+\nu) + \left(\sin^2\theta(\lambda_{\mathrm{a}} + \nu\lambda_{\mathrm{b}})\frac{dL^2}{dT} + \cos^2\theta(\lambda_{\mathrm{b}} + \nu\lambda_{\mathrm{a}})\frac{dL^2}{dT}\right)\right]. \tag{39}$$

can be fitted to $b_1 C_{\mathrm{Debye}} + b_2 \frac{dL^2}{dT}$ where b_1 and b_2 are fit parameters, and $\frac{dL^2}{dT}$ is estimated from Eq. 37. The results of these fits along to measured data of $\frac{df_\theta^2}{dT}$ are shown in Fig. 6. The polar plots of the resulting $b_1(\theta)$ and $b_2(\theta)$ are shown in Fig. 7, which confirm that the thermal contribution to strain does not exhibit significant anisotropic behavior. From Eq. (39), the expected angle dependence of the parameter b_2 is

$$b_2(\theta) = -\left(\frac{E}{4\rho w^2(1-\nu^2)}\right)\left[(\sin^2\theta(\lambda_{\mathrm{a}} + \nu\lambda_{\mathrm{b}}) + \cos^2\theta(\lambda_{\mathrm{b}} + \nu\lambda_{\mathrm{a}})\right], \tag{40}$$

which we use to fit $b_2(\theta)$ in Fig.7 to $A\sin^2\theta + B\cos^2\theta$ where

$$\frac{A}{B} = \frac{\lambda_{\mathrm{a}} + \nu\lambda_{\mathrm{b}}}{\lambda_{\mathrm{b}} + \nu\lambda_{\mathrm{a}}}. \tag{41}$$

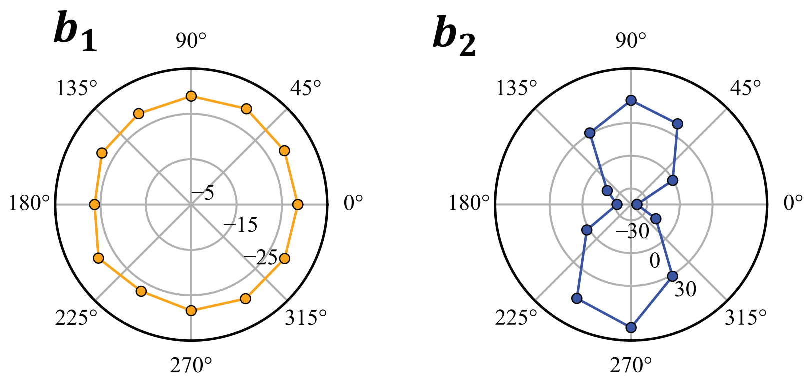

SUPPLEMENTARY FIG. 7. **Polar plot of fit parameters b_1 and b_2:** Polar plot of fit parameters b_1 and b_2 from the fits to df^2/dT shown in Fig. 6.

The fit yields $A/B = -2.062$ and -1.798 for $CoPS_3$ and $A/B = -5.025$ and -8.695 for $FePS_3$.

Supplementary Note 4. ANISOTROPIC RESONANCE FREQUENCY OF $FePS_3$ RESONATORS

Figure 8 shows resonance frequency data measured on $FePS_3$ star-cavity resonators, as presented in Fig. 2 of the main text for $CoPS_3$ and $NiPS_3$ samples. Figure 8a shows the temperature dependence of the resonance frequency of membranes oriented along the a-axis (in red) and b-axis (in blue). Similarly to $CoPS_3$, the opposite strain along a and b arising from spontaneous magnetostriction results in opposite behaviour of the resonance frequency near the transition temperature T_N. The resulting anisotropic response is further illustrated in the map plot of resonance frequency as a function of temperature and angles in Fig. 8b and in the polar plot of $f_{res}(T) - f_{res}(140\,K)$ in Fig. 8c.

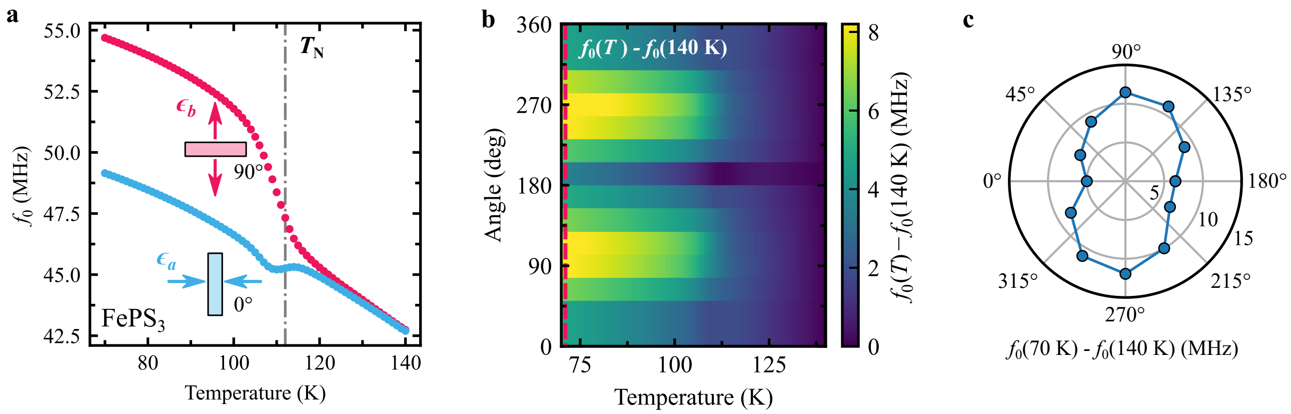

SUPPLEMENTARY FIG. 8. **Angle-resolved resonance frequency data of $FePS_3$ membranes. a** Temperature dependence of f_{res} of a $FePS_3$ rectangular membrane orientated at 0° (blue) and 90° (red) with respect to the b crystallographic axis. Note that $f_{0,0°}$ and $f_{0,90°}$ are proportional to the strain ϵ_a and ϵ_b respectively (see Eq. 2). The dashed-dotted grey line indicates the transition temperature T_N. **b** Resonance frequency difference, $f_{res}(T) - f_{res}(140\,K)$, as a function of angle θ with respect to b-axis and temperature. **c** Polar plot of $f_{res}(T) - f_{res}(140\,K)$ taken along the red dashed line in (b).

Supplementary Note 5. ORDER PARAMETER RELATED FREQUENCY DIFFERENCE $\tilde{f}_b^2 - \tilde{f}_\theta^2$

In the main text, we have shown how to relate the difference $f_b^2 - f_a^2$ to the antiferromagnetic order parameter through the magnetostriction induced strain at the phase transition $\epsilon_{ms,aa} = \lambda_a L^2$ and $\epsilon_{ms,bb} = \lambda_b L^2$. In general (see derivation in Supplementary Note 2), $\tilde{f}_b^2 - \tilde{f}_\theta^2$ is also proportional to L^2, where $\tilde{f}_\theta^2$ is the pretension corrected resonance

frequency of a rectangular cavity oriented at an angle θ with respect to the b-axis. We show this quantity for the $CoPS_3$ and $FePS_3$ star-cavity resonators in Fig. 9a,d by plotting $\tilde{f}_{\mathrm{b}}^2 - \tilde{f}_{\theta}^2$ as a function of angle and temperature in Fig. 9b,e. Figure 9c,f shows the polar plot of $\tilde{f}_{\mathrm{b}}^2 - \tilde{f}_{\theta}^2$ taken along the red dashed line in 9b,e.

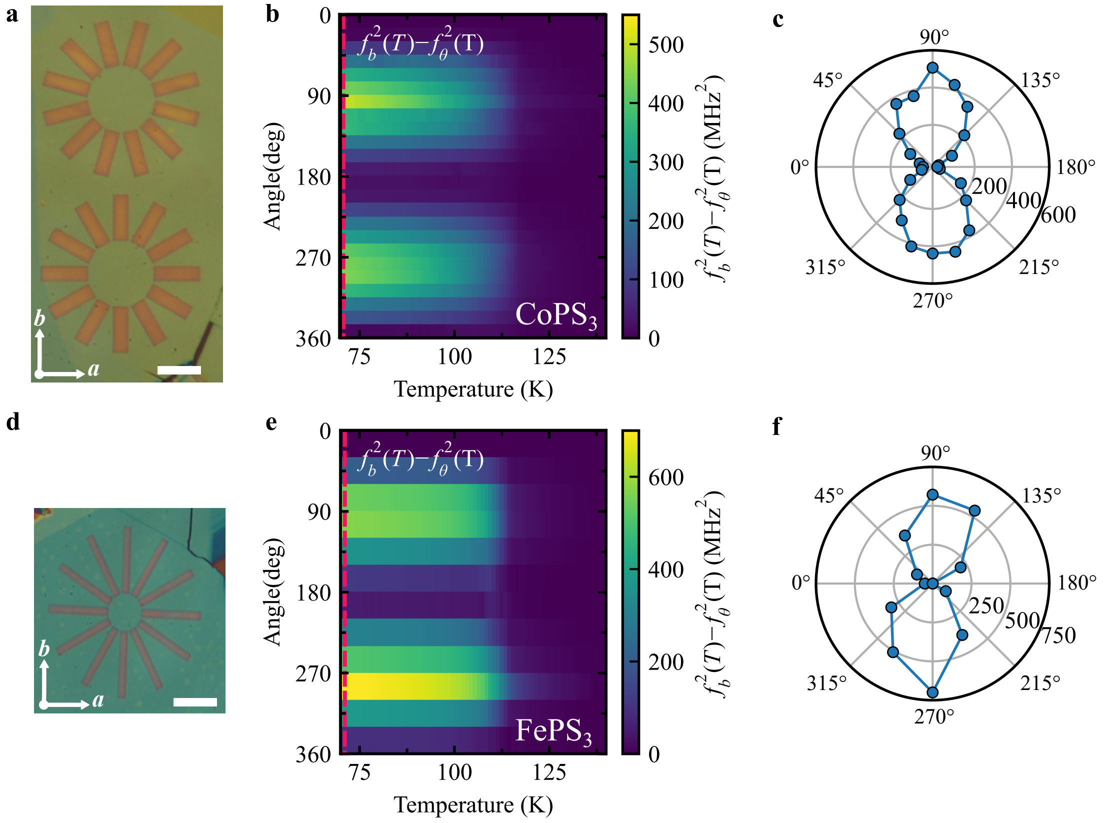

SUPPLEMENTARY FIG. 9. **Angle-resolved data of $f_{\mathrm{b}}^2 - f_{\theta}^2 \propto L^2$ of $CoPS_3$ and $FePS_3$ membranes. a,c** Optical image of the $CoPS_3$ (a) and $FePS_3$ (c) resonators. Scale bar 12 μm. **b** Resonance frequency difference, $f_{\mathrm{b}}^2 - f_{\theta}^2 \propto L^2$, as a function of angle θ and temperature of the $CoPS_3$ sample in (a). **c** Polar plot of $f_{\mathrm{b}}^2 - f_{\theta}^2$ taken along the red dashed line in (b). **e,f** follows the same structure as (b,c) for the $FePS_3$ sample in (c).

This behaviour is observed for the thicker samples ($t > 10$ nm) and it is exploited to have a better estimate of the critical parameters β and T_{N} as discussed in Supplementary Note 6. For thinner resonators every irregularity, like wrinkles or tears, can strongly affect their mode shapes. In some cases, these imperfections can drastically change the resonance frequency of the fundamental mode, as well as its temperature dependence. Therefore, when analysing the critical behaviour of thin flakes, we choose only the most pristine and unaffected membranes out of all fabricated out of a single flake to be sure we are not affected by these irregularities.

Supplementary Note 6. CRITICAL CURVE FIT

To extract critical parameters β and T_{N} shown in Fig. 3 and 4 of the main text, we fit the order parameter related difference $f_{\mathrm{b}}^2 - f_{\mathrm{a}}^2 \propto L^2$ to the power law $A_\theta(1 - T/T_{\mathrm{N}})^{2\beta}$. The experimental determination of critical parameters is often debated due to the difficulty of extracting from one set of data, three strongly correlated parameters, β, T_{N} and A_θ. In addition, finite size effects are known to smear the transition which usually results in a non-zero tail of the order parameter in the disordered state and makes it harder to unambiguously determine the critical temperature. Also, the choice of the temperature interval for the fit is not universal and it is often arbitrary.

In order to have a better estimate of the critical exponents from our experiments, we compute $f_{\mathrm{b}}^2 - f_{\theta}^2$ for all θ in a star and fit the data to $A_\theta(1 - T/T_{\mathrm{N}})^{2\beta}$. For each star, we then calculate the average value and standard deviation of the critical parameters T_{N} and β weighted by the error from the fit $T_{\mathrm{N,err}}$ and β_{err}.

We start with an initial guess, T_{N}^*, for T_{N} by extracting the maximum of the derivative of $f_{\mathrm{b}}^2 - f_{\theta}^2$ with respect to temperature as shown in Fig. 10. We then fit the $A_\theta(1 - T/T_{\mathrm{N}})^{2\beta}$ to $f_{\mathrm{b}}^2 - f_{\theta}^2$ over the range $[\alpha T_{\mathrm{N}}^*, T_{\mathrm{N}}^*]$, for α varying

between $[0.85, 0.95]$ allowing $A, T_{\rm N}, \beta$ to vary. We then define the total error for each α to be $T_{\rm N,err} + \beta_{\rm err}$, where $T_{\rm N,err}$, $\beta_{\rm err}$ are the standard deviation errors of the fit. We then take the extracted $T_{\rm N}, \beta$ with corresponding $T_{\rm N,err}$, $\beta_{\rm err}$ to be ones given the fit corresponding to the α minimizing the total error. We repeat this process for each θ yielding a distribution of $T_{\rm N}, \beta$. We then extract a weighted mean of this distribution as follows:

$$\overline{\beta} = \frac{1}{N} \sum_{\theta} \beta \frac{\beta_{\rm err,min}}{\beta_{\rm err}} \tag{42}$$

Where N is the number of cavity pairs and $\beta_{\rm err,min}$ the $\beta_{\rm err}$ of the pairing with smallest $\beta_{\rm err}$. We then fit a normal distribution with $\overline{\beta}$ as mean to the distribution of β where weigh each β by $\frac{\beta_{\rm err,min}}{\beta_{\rm err}}$, from which we extract the standard deviation. Repeating this process for T_N. The resulting parameters for each sample are listed in Table 50.

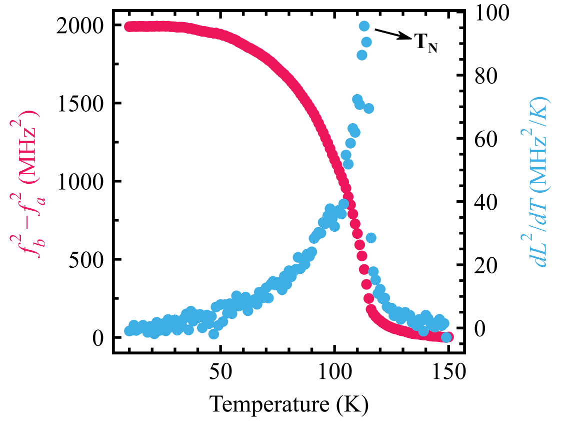

SUPPLEMENTARY FIG. 10. **First estimate of $T_{\rm N}$ from the derivative of the order parameter.**

Material	Sample	t (nm)	β	$T_{\rm N}$ (K)
$CoPS_3$	1	33	0.28 ± 0.017	115.7 ± 0.38
$CoPS_3$	2	52	0.311 ± 0.025	117 ± 0.35
$CoPS_3$	3	52	0.298 ± 0.034	116.1 ± 0.18
$CoPS_3$	4	8	0.195 ± 0.0446	107.8 ± 2.65
$CoPS_3$	5	8.6	0.218 ± 0.002	102.5 ± 1.3
$FePS_3$	1	60	0.208 ± 0.0328	112.7 ± 0.87
$FePS_3$	2	40	0.203 ± 0.03	109.1 ± 0.37
$FePS_3$	3	10	0.194 ± 0.023	110.2 ± 0.48
$FePS_3$	4	7	0.206 ± 0.047	107.9 ± 0.76
$NiPS_3$	1	48	0.218 ± 0.016	150.7 ± 0.7

SUPPLEMENTARY TABLE 50. **Critical exponents of MPS_3 samples.** Critical exponents, β and $T_{\rm N}$, for $CoPS_3$, $FePS_3$ and $NiPS_3$ samples of different thicknesses, extracted following the procedure described in Supplementary Information Supplementary Note 6

[1] Landau, L. D., Pitaevskii, L. P. & Lifshitz, E. M. *Electrodynamics of continuous media*, vol. 8 (Butterworth, New York, 1984), 2 edn.

[2] Landau, L. D. & Lifshitz, E. M. *Theory of Elasticity* (Elsevier, 1986), 3 edn.

[3] Šiškins, M. *et al.* Magnetic and electronic phase transitions probed by nanomechanical resonators. *Nat. Commun.* **11**, 2698 (2020).

[4] Bunch, J. S. *Mechanical and electrical properties of graphene sheets* (Ph.D. thesis, Cornell University Ithaca, NY, 2008).

[5] Šiškins, M. *et al.* Highly anisotropic mechanical and optical properties of 2D layered As_2S_3 membranes. *ACS Nano* **13**, 10845–10851 (2019).
